# Significant CD4, CD8, and CD19 Lymphopenia in Peripheral Blood of Sarcoidosis Patients Correlates with Severe Disease Manifestations

**DOI:** 10.1371/journal.pone.0009088

**Published:** 2010-02-05

**Authors:** Nadera J. Sweiss, Rafah Salloum, Seema Ghandi, Maria-Luisa Alegre, Ray Sawaqed, Maria Badaracco, Kenneth Pursell, David Pitrak, Robert P. Baughman, David R. Moller, Joe G. N. Garcia, Timothy B. Niewold

**Affiliations:** 1 Sarcoidosis and Scleroderma Clinic, Section of Rheumatology, University of Chicago, Chicago, Illinois, United States of America; 2 Section of Rheumatology, University of Chicago, Chicago, Illinois, United States of America; 3 Department of Medicine, University of Chicago, Chicago, Illinois, United States of America; 4 Methodist Hospital, Merrillville, Indiana, United States of America; 5 Section of Infectious Diseases and Global Health, University of Chicago, Chicago, Illinois, United States of America; 6 Division of Pulmonary and Critical Care, University of Cincinnati Medical Center, Cincinnati, Ohio, United States of America; 7 Department of Medicine, Division of Pulmonary and Critical Care Medicine, Johns Hopkins University, Baltimore, Maryland, United States of America; 8 Section of Pulmonary/Critical Care, University of Chicago, Chicago, Illinois, United States of America; Centre de Recherche Public de la Santé (CRP-Santé), Luxembourg

## Abstract

**Background:**

Sarcoidosis is a poorly understood chronic inflammatory condition. Infiltration of affected organs by lymphocytes is characteristic of sarcoidosis, however previous reports suggest that circulating lymphocyte counts are low in some patients with the disease. The goal of this study was to evaluate lymphocyte subsets in peripheral blood in a cohort of sarcoidosis patients to determine the prevalence, severity, and clinical features associated with lymphopenia in major lymphocyte subsets.

**Methodology/Principal Findings:**

Lymphocyte subsets in 28 sarcoid patients were analyzed using flow cytometry to determine the percentage of CD4, CD8, and CD19 positive cells. Greater than 50% of patients had abnormally low CD4, CD8, or CD19 counts (p<4×10^−10^). Lymphopenia was profound in some cases, and five of the patients had absolute CD4 counts below 200. CD4, CD8, and CD19 lymphocyte subset counts were significantly correlated (Spearman's rho 0.57, p = 0.0017), and 10 patients had low counts in all three subsets. Patients with severe organ system involvement including neurologic, cardiac, ocular, and advanced pulmonary disease had lower lymphocyte subset counts as a group than those patients with less severe manifestations (CD4 p = 0.0043, CD8 p = 0.026, CD19 p = 0.033). No significant relationships were observed between various medical therapies and lymphocyte counts, and lymphopenia was present in patients who were not receiving any medical therapy.

**Conclusions/Significance:**

Significant lymphopenia involving CD4, CD8, and CD19 positive cells was common in sarcoidosis patients and correlated with disease severity. Our findings suggest that lymphopenia relates more to disease pathology than medical treatment.

## Introduction

Sarcoidosis is a systemic inflammatory disease characterized by noncaseating granulomas which consist of CD4^+^ T-cells and macrophages surrounded by CD8^+^ T-cells [1]. This inflammation can affect any number of organs, including the lung, liver, heart, skin, and nervous system. The etiology of sarcoidosis is unknown, but likely involves genetic factors, which interact with environmental exposures to result in disease susceptibility [2]. The immune dysregulation in sarcoidosis is classically manifested by hypergammaglobulinemia and paradoxical cutaneous anergy to particular antigens such as tuberculin protein [3]. This paradox of anergy co-existing with an inflammatory condition remains poorly understood.

Lymphopenia occurs in over 50% of sarcoidosis patients and is associated with chronic disease [4], [5], [6]. While early studies have suggested that sarcoidosis-associated lymphopenia is due to T-cell depletion [7], few studies have focused on the various peripheral blood lymphocyte subsets in sarcoidosis. Miyara and colleagues investigated the presence and function of regulatory T cells in the lung and blood of patients with sarcoidosis. It was demonstrated that CD4^+^CD25^bright^FoxP3^+^ cells accumulated in the periphery of sarcoid granulomas, in BAL washings, and in peripheral blood of patients with active disease. These cells effectively suppressed the proliferation of effector cells but not their production of TNF. This may explain the paradoxical state of T cell anergy in these patients that is associated with active local inflammation. However, to our knowledge, detailed examination of the various lymphocyte subsets in the peripheral blood in sarcoidosis patients has not been described. Thus, the goal of this study was to evaluate lymphocyte subsets in peripheral blood in a cohort of sarcoidosis patients followed at the University of Chicago Rheumatology Clinic to determine the prevalence, severity, and clinical features associated with lymphopenia in major lymphocyte subsets.

## Materials and Methods

We performed a retrospective review of the laboratory records for lymphocyte subset testing in sarcoidosis patients at the University of Chicago Hospital between November 2006 and January 2008. Our analysis was limited to individuals who received full testing by flow cytometry and included the following cell counts: Absolute CD4 (515–1642 cells/µL), CD8(212–887 cells/µL) and CD19 (103–581 cells/µL). The medical records of patients and controls were reviewed according to the study approved by the University of Chicago Institutional Review Board. Because this was a retrospective chart review, patient consent was waived by the Institutional Review Board as the study approved by the Institutional Review Board required no therapeutic intervention. Twenty-eight patients were identified. Inclusion criteria were patients with biopsy-proven sarcoidosis who attended the University of Chicago Sarcoidosis Center for new or return appointments and had full lymphocyte subset data available.

Clinical data were obtained by the primary study physician (NJS) and abstracted to include clinical manifestations related to sarcoidosis, demographics, and medical therapy. Lymphocyte subset data in the sarcoidosis cohort were compared to 100 healthy individuals who were tested by the clinical laboratory for assay standardization. Results were also compared between groups of patients defined by disease manifestations or medical treatment. Severe organ system involvement was defined as chronic sarcoidosis with at least one of the following disease manifestations: neurologic, cardiac, ocular, or advanced pulmonary disease defined as forced vital capacity (FVC)<40% of predicted. Patients with severe sarcoidosis typically require corticosteroid-sparing agents secondary to corticosteroid failure.

### Flow Cytometry

CD4, CD8, and CD19 cells were counted in whole blood using flow cytometry techniques at the University of Chicago Clinical Laboratory following standard protocols with fluorescently labeled antibodies. The proportion of cells positive for CD4, CD8, and CD19 was multiplied by the total number of lymphocytes, and cell counts were reported as number of cells per microliter (cells/µL). Normal ranges for CD4, CD8, and CD19 cell populations were derived from the healthy donor cohort used to standardize the assay. Low count was defined as results below the lower level of normal for CD4 (515–1642 cells/µL), CD8 (212–887 cells/µL) and CD19 (103–581 cells/µL).

### Statistical Analysis

Fisher's exact test was used to compare categorical data, and quantitative comparisons were performed using an unpaired t-test or Mann-Whitney non-parametric t-test as appropriate depending upon whether the data were normally distributed as assessed by the D'Agostino and Pearson omnibus normality test. P-values shown are all two-sided, and uncorrected for multiple comparisons.

## Results

### Patient Characteristics

In this cohort of 28 sarcoidosis patients, 18 were African-American, and ten were European-American. The mean age was 51.6 years, with a wide spectrum of organ system involvement (Table 1). Seven patients (25%) had cardiac and 7 had skin involvement, 4 patients (14%) had clinically significant lung involvement, 4 (14%) had neurologic involvement, and 6 patients (21%) had liver involvement. A total of 24/28 patients in this cohort (86%) required therapy.

**Table 1:** Demographic and Clinical Information for the Cohort.

Total # of patients	28
# of African-American	18
# of European-American	10
Average age of patient (yrs)	51.6
Number of pt with severe disease No. (%)	18 (64%)
Heart Involvement No. (%)	7 (25%)
Skin Involvement No. (%)	7 (25%)
Lung Involvement No. (%)	4 (14%)
Liver Involvement No. (%)	6 (21%)
Neurologic Involvement No. (%)	4 (14%)
Number of patients requiring therapy No. (%)	26 (86%)

### Significant CD4, CD8, and CD19 Lymphopenia Was Present in Sarcoidosis Patients

**Figure 1:**
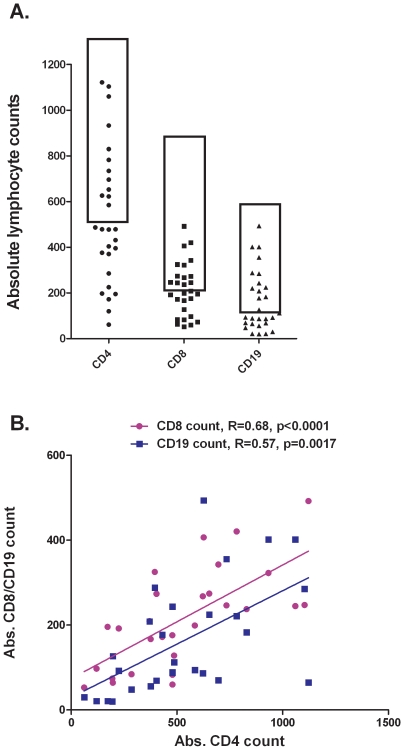
Absolute counts and correlation between CD4, CD8, and CD19 lymphocytes in sarcoidosis. A) Peripheral blood lymphocyte measurements show that 16 patients (57%) had lower than normal CD4 counts, 15 patients (54%) had lower than normal CD8 counts, and 15 patients (54%) had lower than normal CD19 counts. The box indicates the normal range for cell counts (95% of normal control subjects fell within this normal range) (p<4×10^−10^). B) Correlation between lymphocyte counts in sarcoidosis patients. CD4∶CD8 correlation shown in purple, and CD4∶CD19 shown in blue, with Spearman's rho and p-value indicated for each correlation.

The majority of sarcoidosis patients exhibited reduced lymphocyte subset counts, including CD4 (57%), CD8 (54%), and CD19 cell counts (54%) as compared to healthy controls (Figure 1A, p<4×10^−10^). Only eight patients (29%) demonstrated values within the normal range for all three lymphocyte subsets. Lymphopenia was profound in some cases, and five of the patients had absolute CD4 counts below 200. There were no significant differences by gender or ethnicity (European vs. African-American ancestry) in any of the lymphocyte subset counts. CD4, CD8, and CD19 lymphocyte counts were significantly correlated (Spearman's rho 0.57, p = 0.0017, Figure 1B), and 10 patients (36%) exhibited reduced lymphocyte counts in all three subsets.

### Lymphopenia in Sarcoidosis Patients Is Not Related to Medical Therapy but Instead to Severity of Disease

**Figure 2:**
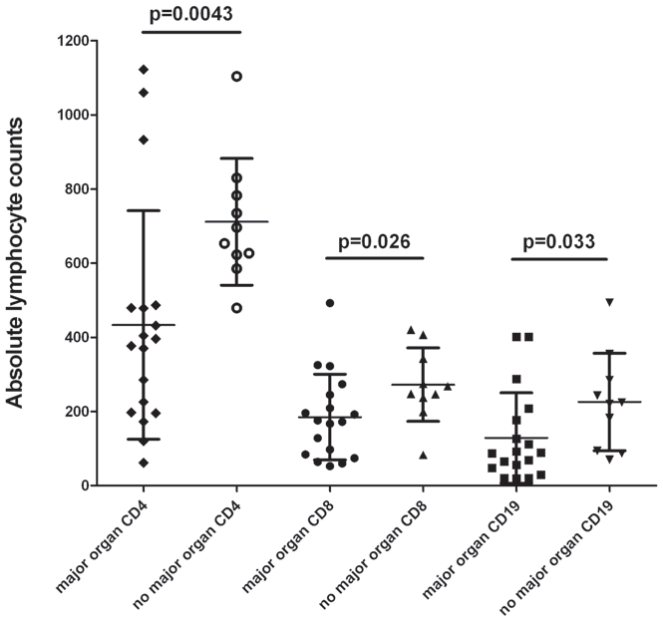
Lymphocyte counts in patients with severe organ system involvement vs. those without severe organ system involvement. Severe organ system involvement is defined as outlined in the methods.

Reductions in lymphocyte subset counts were not significantly different in patients who were taking disease-modifying anti-rheumatic drugs (DMARDs, including methotrexate, azathioprine, leflunomide and mycophenolate mofetil) (n = 14), prednisone (n = 18) or tumor necrosis factor alpha (TNF-α) inhibitors (n = 6) (p>0.5, data not shown). Four patients had not been taking any medical therapy, and three of these patients had abnormally low lymphocyte counts. Patients with severe organ system involvement as defined in the Methods had lower lymphocyte subset counts as a group than those patients who did not have these disease manifestations (CD4 p = 0.0043, CD8 p = 0.026, CD19 p = 0.033, Figure 2).

## Discussion

This study provides the first report of frequent and severe CD4, CD8, and CD19 lymphopenia in sarcoidosis patients to our knowledge. All lymphocyte subsets were highly correlated with each other, suggesting that many patients have severe overall T- and B-cell lymphopenia in peripheral blood. Lymphopenia was frequently present in patients who were not receiving any medical therapy, and while immunosuppressive therapies may reduce lymphocyte numbers in the peripheral blood [8], we found that there were no significant relationships between specific medical therapies and lymphocyte counts. In contrast, severe organ system involvement was associated with lower lymphocyte subset counts, suggesting that lymphopenia may relate more to disease pathology than medical therapy. This finding could suggest that lymphocytes are depleted in peripheral blood due to increased infiltration of target organs. In one study of pulmonary sarcoidosis patients undergoing bronchoalveolar lavage (BAL), there was a was a significant relationship between an increase in the number of CD4 positive T cells in the BAL fluid and a decrease in the absolute number of CD4 positive T cells in the peripheral blood [9]. Alternatively, lymphopenia could represent either suppression of lymphogenesis due to cytokines or other influences, or increased peripheral destruction due to activation-driven cell death or other mechanisms. Longitudinal follow up studies would be of high interest to determine whether lymphopenia is corrected with therapy, as other disease manifestations are effectively treated. Such results would further strengthen the hypothesis that lymphopenia underlies disease pathology rather than being a result of generalized immunosuppression.
