# Correction: Significant CD4, CD8, and CD19 Lymphopenia in Peripheral Blood of Sarcoidosis Patients Correlates with Severe Disease Manifestations

**DOI:** 10.1371/annotation/a75007e1-492a-4bcb-80a8-28b4d432c099

**Published:** 2010-02-24

**Authors:** Nadera J. Sweiss, Rafah Salloum, Seema Gandhi, Maria-Luisa Alegre, Ray Sawaqed, Maria Badaracco, Kenneth Pursell, David Pitrak, Robert P. Baughman, David R. Moller, Joe G. N. Garcia, Timothy B. Niewold

The third author's name was spelled incorrectly. The correct name is Seema Gandhi. The correct citation is: Sweiss NJ, Salloum R, Gandhi S, Alegre M-L, Sawaqed R, et al. (2010) Significant CD4, CD8, and CD19 Lymphopenia in Peripheral Blood of Sarcoidosis Patients Correlates with Severe Disease Manifestations. PLoS ONE 5(2): e9088. doi:10.1371/journal.pone.0009088

